# Non-contiguous finished genome sequence and description of *Bartonella florenciae* sp. nov.

**DOI:** 10.4056/sigs.4358060

**Published:** 2013-10-10

**Authors:** Oleg Mediannikov, Khalid El Karkouri, Catherine Robert, Pierre-Edouard Fournier, Didier Raoult

**Affiliations:** 1 URMITE, Aix-Marseille Université, Faculté de médecine, Marseille, France; 2 URMITE, Campus commun UCAD-IRD d'Hann, Dakar, Senegal; 3 King Fahad Medical Research Center, King Abdul Aziz University, Jeddah, Saudi Arabia

**Keywords:** *Bartonella florenciae*, genome, France, shrew, *Crocidura russula*, taxonogenomics

## Abstract

*Bartonella florenciae* sp. nov. strain R4^T^ is the type strain of *B. florenciae* sp. nov., a new species within the genus *Bartonella*. This strain, whose genome is described here, was isolated in France from the spleen of the shrew *Crocidura russula*. *B. florenciae* is an aerobic, rod-shaped, Gram-negative bacterium. Here we describe the features of this organism, together with the complete genome sequence and its annotation. The 2,010,844 bp-long genome contains 1,909 protein-coding and 46 RNA genes, including two rRNA operons.

## Introduction

*Bartonella* is the monotypic genus of the family *Bartonellaceae*, classified among the α-*Proteobacteria*. To date, 29 *Bartonella* species have been officially validated [1,2], and many isolates have yet to be described [3,4]. Species of this genus share many general characteristics. They are small (usually less than 1μm), Gram-negative, pleomorphic coccobacilli. All members of the genus are fastidious and grow slowly *in vitro*. These bacteria are facultatively intracellular and use hemotrophy (infection of erythrocytes) as a parasitic strategy [5,6]. *Bartonella* species infect a wide range of animal species, including domestic animals such as cats, dogs, rodents, rabbits and cattle as well as a diverse group of wild animals including wildcats, coyotes, deer, elks, foxes, insectivores, bats, etc. The epidemiological cycle of bartonellae consists of a reservoir host with a chronic intravascular infection and sustained bacteremia, and a vector that transfers the bacteria from the reservoir to a susceptible host. Thus, bartonellae may be identified and isolated from a number of blood-sucking arthropods associated with the vertebrate hosts of bacteria. Proven vectors include sandflies, hippoboscids, fleas, soft and hard ticks, lice and mites. Many *Bartonella* species are associated with human diseases. *Bartonella bacilliformis*, *B. quintana* and *B. henselae* are relatively common human pathogens. Other less common pathogenic species include rodent-associated species, such as *B. elizabethae*, *B. grahamii* and *B. vinsonii* [7-9]. The shrew *Crocidura russula* is an insectivore mammal in which a *Bartonella* strain was once identified in Korea [10]. To date, only one officially recognized *Bartonella* species, *B. talpae*, was detected in insectivores. However, no type strain is available for this species and its genetic characterization was not achieved [1,11].

In 2003, La Scola *et al.* proposed a multilocus sequence analysis based on 4 genes and one intergenic spacer as a tool for the description of new *Bartonella* species [12]. Two of these markers, *i.e.*, *gltA* and *rpoB*, were particularly discriminatory, with new *Bartonella* isolates considered as new species if they exhibit <96.0% and <95.4% sequence identity with other validated species for the 327- and 825-bp fragments of the *gltA* and *rpoB* genes, respectively. This strategy being congruent with the “gold-standard” DNA–DNA reassociation for several bacterial genera [13], these criteria have since been regularly applied for the description of new *Bartonella* species [2,14].

In this study, we used both the genetic criteria of La Scola *et al*. and the genome sequence, as well as the main phenotypic characteristics of strain R4^T^ to present a summary classification and a set of features for *B. florenciae* sp. nov. strain R4^T^(DSM 23735 = CSUR B627). These characteristics support the circumscription of the *B. florenciae* sp. nov.

## Classification and features

In February 2010, an adult *Crocidura russula* shrew was found dead without evident signs of trauma near the parking lot of the calanque d’En-Vau close to Marseille, France. The shrew was brought to the laboratory where the cardiac blood and the organs (spleen, liver and brain) were collected. The organs ground in Rinaldini solution were inoculated on Columbia agar (BioMerieux, Marcy l'Etoile, France) as previously described [15]. Strain R4 (Table 1) was obtained from the spleen following a 7-day incubation at 37°C in 5% CO_2_-enriched atmosphere on Columbia agar. Three other morphologically and genetically indistinguishable strains were isolated from the blood, brain and liver from the same shrew.

**Table 1:** Classification and general features of *Bartonella florenciae* strain R4^T^.

**MIGS ID**	**Property**	**Term**	**Evidence code^a^**
		Domain *Bacteria*	TAS [16]
		Phylum *Proteobacteria*	TAS [17]
		Class *Alphaproteobacteria*	TAS [18,19]
	Current classification	Order *Rhizobiales*	TAS [19,20]
		Family *Bartonellaceae*	TAS 21-23]
		Genus *Bartonella*	TAS [21,22,24-26]
		Species *Bartonella florenciae*	IDA
		Type strain R4^T^	IDA
	Gram stain	Negative	IDA
	Cell shape	Rod	IDA
	Motility	Not motile	IDA
	Sporulation	Nonsporulating	IDA
	Temperature range	Mesophilic	IDA
	Optimum temperature	37°C	IDA
MIGS-6.3	Salinity	Growth in BHI medium + 5% NaCl	IDA
MIGS-22	Oxygen requirement	Aerobic	IDA
	Carbon source	Unknown	IDA
	Energy source	Unknown	IDA
MIGS-6	Habitat	*Crocidura russula*	IDA
MIGS-15	Biotic relationship	Facultative intracellular	IDA
	Pathogenicity	Unknown	
	Biosafety level	3	
MIGS-14	Isolation	Spleen of the shrew *Crocidura russula*	IDA
MIGS-4	Geographic location	France	IDA
MIGS-5	Sample collection time	February 2010	IDA
MIGS-4.1	Latitude	43.216667	IDA
MIGS-4.2	Longitude	5.5	IDA
MIGS-4.3	Depth	Surface	IDA
MIGS-4.4	Altitude	40 m above sea level	IDA

^a^ Evidence codes - IDA: Inferred from Direct Assay; TAS: Traceable Author Statement (*i.e*., a direct report exists in the literature); NAS: Non-traceable Author Statement (*i.e*., not directly observed for the living, isolated sample but based on a generally accepted property for the species or anecdotal evidence). Evidence codes come from the Gene Ontology project [27]. If the evidence is IDA, then the property was directly observed for a live isolate by one of the authors or an expert mentioned in the acknowledgements.

**Figure 1:**
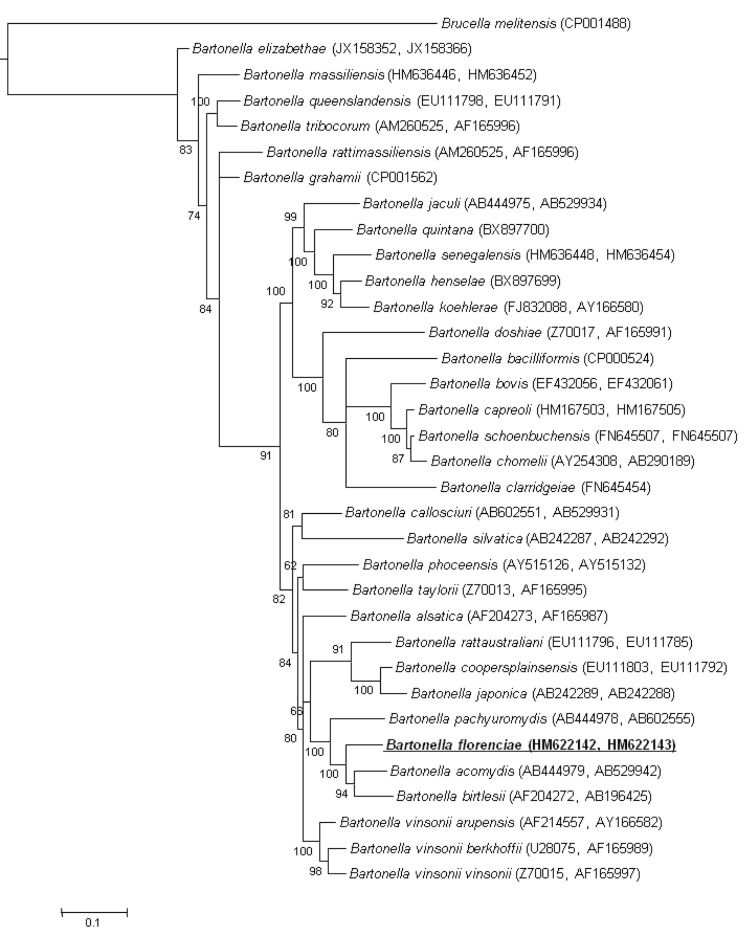
Phylogenetic tree highlighting the position of *B. florenciae* strain R4^T^ relative to other type strains within the genus *Bartonella*. Concatenated *gltA* and *rpoB* sequences were aligned using CLUSTALW and phylogenetic inferences obtained using Bayesian phylogenetic analysis [32] with the TOPALi 2.5 software (Biomathematics and Statistics Scotland, Edinburgh, UK) with the integrated MrBayes application [33] with the following substitution models: HKY for the first codon position, GTR+Г for the second codon position and GTR+Г+I for the third codon position. GenBank accession numbers are indicated in parentheses as (*gltA*, *rpoB*). Numbers at the nodes are bootstrap values obtained by repeating the analysis 100 times to generate a majority consensus tree. There were a total of 1,044 positions in the final dataset. The scale bar indicates a 10% nucleotide sequence divergence.

In addition to *gltA* and *rpoB* partial gene sequencing, we also sequenced the intergenic transcribed spacer (ITS) along with the 16S rRNA and *ftsZ* genes as previously described [10,28-31]. The ITS and 16S rRNA of strain R4T exhibited nucleotide sequence similarities of 63.8% and 99.4% with those of Bartonella tribocorum strain CIP 105476, respectively (GenBank accession number AF312505 and NR_074354, respectively); 94.4% with *Bartonella birtlesii* strain IBS 325 for *ftsZ* (AM690313), 92.6% with *Bartonella acomydis* strain KS2-1 for *rpoB* (AB529942) and 90.7% with *Bartonella taylorii* strain M6 for *gltA* (Z70013). Phylogenetically, strain R4^T^ formed a separate branch among the rodent-associated species (Figure 1).

**Figure 2:**
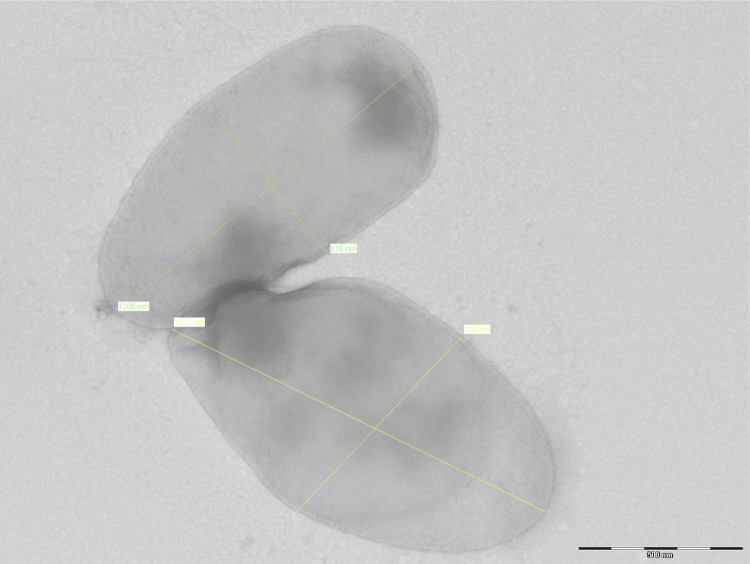
Transmission electron micrograph of *B. florenciae* strain R4^T^, using a Morgagni 268D (Philips) transmission electron microscope at an operating voltage of 60 kV. The scale bar represents 500 nm.

Different growth temperatures (32, 37, 42°C) were tested. Growth only occurred at 37°C in 5% CO_2_ atmosphere. Colonies were gray, opaque and 0.3 mm to 1 mm in diameter on blood-enriched Columbia agar. Cells grown on agar are Gram-negative and have a mean length and width of 1.39± 0.3 µm and 0.63±0.1 µm, respectively, by electron microscopy (Figure 2). No flagella or pili were observed.

Strain R4^T^ exhibited neither catalase nor oxidase activities. Biochemical characteristics were assessed using an Anaerobe Identification Test Panel AN MicroPlate™ (Biolog Inc., Hayward, CA, USA). None of the 95 biochemical tests available (including D-mannose, D-fructose and D-galactose) were positive. Similar profiles were previously observed for other *Bartonella* species [14].

**Figure 3:**
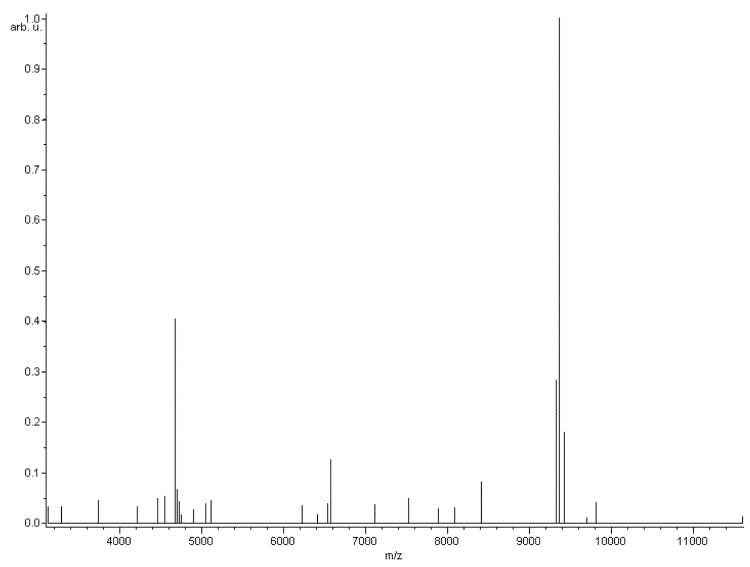
Reference mass spectrum from *B. florenciae* strain R4^T^. Spectra from 12 individual colonies were compared and a reference spectrum was generated.

**Figure 4:**
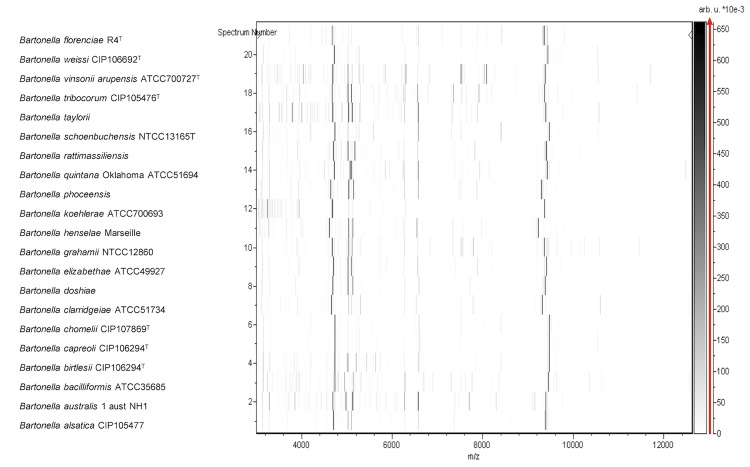
Gel view comparing *B. florenciae* sp. nov*.,* strain R4^T^ with other members of the genus *Bartonella*. The gel view displays the raw spectra of all loaded spectrum files arranged in a pseudo-gel like fashion. The x-axis records the m/z value. The left y-axis displays the running spectrum number originating from subsequent spectra loading. The peak intensity is expressed by a Gray scale scheme code. The color bar and the right y-axis indicate the relation between the color a peak is displayed with and the peak intensity in arbitrary units.

Matrix-assisted laser desorption/ionization time-of-flight (MALDI-TOF) mass spectrometry protein analysis was carried out as previously described using a Microflex spectrometer (Bruker Daltonics, Leipzig, Germany) [34]. Twelve individual colonies were deposited on a MTP 384 MALDI-TOF target plate (Bruker). Each smear was overlaid with 2 μL of matrix solution (a saturated solution of alpha-cyano-4-hydroxycinnamic acid) in 50% acetonitrile/2.5% trifluoroacetic acid, and allowed to dry for five minutes. The twelve R4^T^ spectra were imported into the MALDI BioTyper software (version 2.0, Bruker) and analyzed by standard pattern matching (with default parameter settings) against the main spectra of 4,613 bacteria, including 241 spectra from 20 validly named *Bartonella* species, used as reference data in the BioTyper database. A score enabled the presumptive identification and discrimination of the tested species from those in a database: a score > 2 with a validated species enabled the identification at the species level; and a score < 1.7 did not enable any identification. For strain R4^T^, no significant score was obtained, suggesting that our isolate was not a member of any known species (Figures 3 and 4). The gel view shows the spectrum differences with other species within the *Bartonella* genus (Figure 4).

## Genome sequencing information

### Genome project history

The organism was selected for sequencing on the basis of the similarity of its 16S rRNA, ITS, *ftsZ*, *gltA* and *rpoB* to other members of the genus *Bartonella*. Nucleotide sequence similarity levels of these genes suggested that strain R4^T^ represents a new species within the genus *Bartonella*. It was the eleventh genome of a *Bartonella* species and the first genome of *Bartonella florenciae* sp. nov. A summary of the project information is shown in Table 2. The GenBank accession number is CALU00000000 and consists of 62 contigs (14 scaffolds). Table 3 shows the project information and its association with MIGS version 2.0 compliance.

**Table 2:** Project information

**MIGS ID**	**Property**	**Term**
MIGS-31	Finishing quality	High-quality draft
MIGS-28	Libraries used	One paired-end 3-kb library
MIGS-29	Sequencing platforms	454 GS FLX Titanium
MIGS-31.2	Fold coverage	36x
MIGS-30	Assemblers	Newbler version 2.5.3
MIGS-32	Gene calling method	Prodigal
	EMBL ID	CALU00000000
	EMBL Date of Release	August, 17, 2012
MIGS-13	Project relevance	Biodiversity of the *Crocidura russula* microbial flora

**Table 3:** Nucleotide content and percentage of the genome

**Attribute**	**Value**	**% of total^a^**
Genome size (bp)	2,010,844	100
DNA coding region (bp)	1,624,868	80.8
DNA G+C content (bp)	774,294	38.5
Total genes	1,955	100
RNA genes	46	2.35
Protein-coding genes	1,909	100
Protein with predicted function	1,135	60
Genes assigned to COG	1,328	69.4
Genes with peptide signal	84	4.4
Genes with transmembrane helices (≥3)	193	10

^a^The total is based on either the size of the genome in base pairs or the total number of protein coding genes in the annotated genome.

### Growth conditions and DNA isolation

*B. florenciae* sp. nov. strain R4^T^ (DSM 23735, CSUR B627) was grown on 5% sheep blood-enriched Columbia agar at 37°C in a 5% CO_2_ atmosphere. Four Petri dishes were spread and resuspended in 3×100 μl of G2 buffer (EZ1 DNA Tissue kit, Qiagen). A first mechanical lysis was performed by glass powder on the Fastprep-24 device (Sample Preparation system; MP Biomedicals, USA) using 2×20-second cycles. DNA was then treated with 2.5 μg/μL lysozyme (30 minutes at 37°C) and extracted through the BioRobot EZ 1 Advanced XL (Qiagen). The DNA was then concentrated and purified on a Qiamp kit (Qiagen). The yield and concentration were measured by the Quant-it Picogreen kit (Invitrogen) on the Genios_Tecan fluorometer at 131 ng/μl.

### Genome sequencing and assembly

DNA (5 μg) was mechanically fragmented on a Hydroshear device (Digilab, Holliston, MA, USA) with an enrichment size of 3-4 kb. The DNA fragmentation was visualized using the Agilent 2100 BioAnalyzer on a DNA labchip 7500 with an optimal size of 3.375 kb. The library was constructed according to the 454 GS FLX Titanium paired-end protocol. Circularization and nebulization were performed and generated a pattern with an optimal at 622 bp. After PCR amplification over 17 cycles followed by double size selection, the single-stranded paired-end library was then quantified with the BioAnalyzer on a DNA labchip RNA pico 6000 at 179 pg/μL. The library concentration equivalence was calculated as 1E+08 molecules/μL. The library was stored at -20°C until further use. The library was clonally amplified with 1.5 cpb in 3 emPCR reactions with the GS Titanium SV emPCR Kit (Lib-L) v2 (Roche). The yield of the 1.5 cpb emPCR was determined to be 8.8%, in the 5 to 20% range recommended in the Roche procedure. Approximately 790,000 beads were loaded on a ¼ region on the GS Titanium PicoTiterPlate PTP Kit 70×75 and sequenced with the GS FLX Titanium Sequencing Kit XLR70 (Roche). The run was analyzed on the cluster through the gsRunBrowser and Newbler assembler (Roche). A total of 232,038 passed filter wells were obtained and generated 72.01 Mb of DNA sequence with an average read length of 310 bp.

The passed filter sequences were assembled using Newbler with 90% identity and 40 bp as overlap. The final assembly identified 14 scaffolds and 62 large contigs (>1.5kb) which corresponds to 36× as an equivalence genome.

### Genome annotation

Coding sequences (CDSs) were predicted using PRODIGAL with default parameters [35], but predicted ORFs were excluded if they spanned a sequencing gap region. The functional annotation of protein sequences was performed against the non-redundant GenBank database using BLASTP and functional categories of these proteins was searched against the Clusters of Orthologous Groups (COG) database using COGNITOR [36]. The prediction of RNAs genes, i.e., rRNAs, tRNAs and other RNAs was carried out using RNAmmer [37] and ARAGORN [38] algorithms. The transmembrane helices and signal peptides were identified using TMHMM [39] and SignalP [40] tools, respectively.

## Genome properties

**Figure 5:**
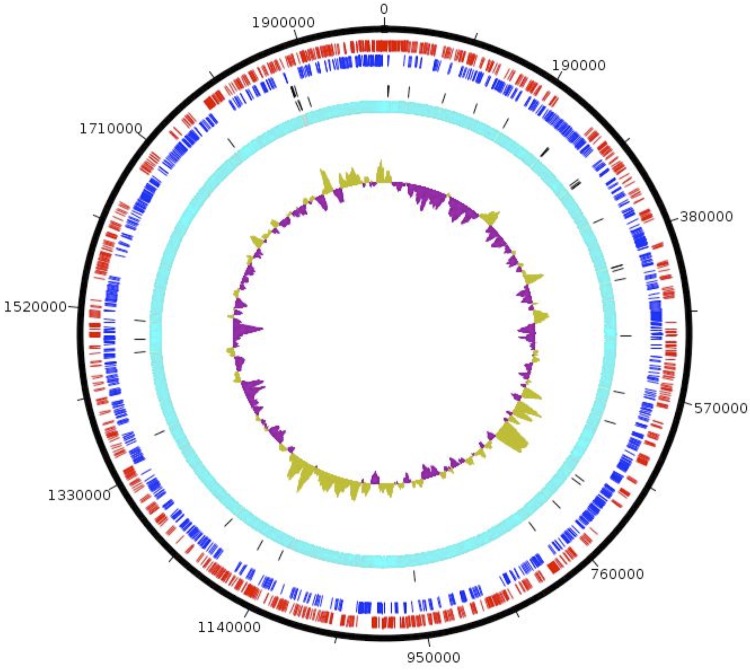
Graphical circular map of the chromosome. From the outside in, the outer two circles show open reading frames oriented in the forward and reverse directions, respectively. The third circle shows rRNA genes. The inner-most circle shows GC skew, purple and olive indicating negative and positive values, respectively.

The genome is 2,010,844 bp long (one chromosome, one plasmid) with a 38.5% GC content (Table 3, Figure 5). Of the predicted genes, 1,909 were protein-coding genes, and 46 were RNAs including two rRNA operons. The plasmid was 25 kb-long and had a total of 28 genes. A total of 1,135 genes (60%) were assigned a putative function. The remaining genes were annotated as either hypothetical proteins or proteins of unknown functions. The distribution of genes into COGs functional categories is presented in Table 4. The properties and the statistics of the genome are summarized in Tables 3 and 4.

**Table 4:** Number of genes associated with the 25 general COG functional categories^†^.

**Code**	**Value**	**%age**	**Description**
J	141	7.43	Translation
A	0	0	RNA processing and modification
K	71	3.74	Transcription
L	98	5.17	Replication, recombination and repair
B	0	0	Chromatin structure and dynamics
D	25	1.32	Cell cycle control, mitosis and meiosis
Y	0	0	Nuclear structure
V	19	1.00	Defense mechanisms
T	39	2.06	Signal transduction mechanisms
M	95	5.01	Cell wall/membrane biogenesis
N	9	0.47	Cell motility
Z	0	0	Cytoskeleton
W	7	0.37	Extracellular structures
U	85	4.48	Intracellular trafficking and secretion
O	74	3.90	Posttranslational modification, protein turnover and chaperones
C	77	4.06	Energy production and conversion
G	62	3.27	Carbohydrate transport and metabolism
E	129	6.80	Amino acid transport and metabolism
F	46	2.42	Nucleotide transport and metabolism
H	59	3.11	Coenzyme transport and metabolism
I	42	2.21	Lipid transport and metabolism
P	75	3.95	Inorganic ion transport and metabolism
Q	14	0.74	Secondary metabolites biosynthesis, transport and catabolism
R	189	9.96	General function prediction only
S	134	7.06	Function unknown
X	581	30.63	Not in COGs

^†^ The total is based on the total number of protein coding genes in the annotated genome.

## Comparison with the *Bartonella tribocorum* genome

Compared to *B. tribocorum* strain CIP105476 (GenBank accession number NC_010161), *B. florenciae* strain R4^T^ had a much smaller genome (2,010,844 and 2,619,061 bp, respectively), less genes (1,955 and 2,135 genes, respectively) and a lower G+C content (38.5% and 38.8%, respectively). Comparative genomics of the proteomes of these bacteria showed that 188 protein-coding genes present in *B. florenciae* were absent or present as pseudogenes in *B. tricoborum*. These included genes encoding the multidrug resistance efflux pump VceA protein, phage proteins, SAM-dependent methyltransferase, tolA protein, transcriptional repressor Arc, Cytosine-specific methyltransferase NlaX, Glycoside hydrolase, conjugal transfer protein TraC/D, major facilitator superfamily (MFS) proteins, NADPH-dependent FMN reductase, lytic transglycosylase, mccB proteins, transcriptional regulator proteins, membrane protein, D-isomer specific 2-hydroxyacid dehydrogenase, putative phosphoribosylglycinamide synthetase and putative type II restriction endonuclease as well as several hypothetical proteins.

## Conclusion

On the basis of phenotypic, phylogenetic and genomic analyses, we formally propose the creation of *Bartonella florenciae* sp. nov. that contains strain R4^T^. This bacterium has been isolated in France.

### Description of *Bartonella florenciae* sp. nov.

*Bartonella florenciae* (flo.ren´ci.ae. N.L. gen. fem. n. *florenciae* of Florence, named in honor of Florence Fenollar, the prominent French microbiologist who found the *Crocidura russula* shrew from which the type strain was isolated).

Colonies are opaque, grey, and 0.5 to 1.0 mm in diameter on blood-enriched Columbia agar. Cells are rod-shaped without flagellae. Length and width are 1.39 ± 0.3 µm and 0.63 ± 0.1 µm, respectively. Growth is achieved at 37°C in aerobic atmosphere enriched with 5% CO_2_. Cells stain Gram-negative, are non-endospore-forming, and are not motile. Catalase and oxidase activities are absent. Using the Anaerobe Identification Test Panel AN MicroPlate, no biochemical activity is observed.

The genome is 2,010,844-bp long (one chromosome and one plasmid) and contains 1,909 protein-coding and 46 RNA genes, including two rRNA operons. The G+C content is 38.5%. Sequences from the ITS, 16S rRNA, *ftsZ*, *rpoB* and *gltA* genes, and the genome are deposited in GenBank under accession numbers HM622140, HM622139, HM622141, HM622143, HM622142 and CALU00000000, respectively. The type strain R4^T^ (DSM 23735, CSUR B627) was isolated from a *C. russula* shrew found dead in calanque d’En-Vau near Marseille, France.
